# Muse-like stem cell therapy for curing chronic diseases in geriatric feline and canine

**DOI:** 10.3389/fvets.2026.1708295

**Published:** 2026-03-18

**Authors:** Yu Chen, Wataru Otsubo, Aoli Li, Hirofumi Hagino, Aijie Liu, Haishi Fan, Chenwen Huang

**Affiliations:** 1 Department of Clinical Research Centre, Shanghai Sixth People's Hospital Affiliated to Shanghai Jiao Tong University School of Medicine, Shanghai, China; 2 Shanghai Wholesomebio Technology Co., Ltd., Shanghai, China; 3 Shanghai Ainikang Veterinary Company Limited, Shanghai, China; 4 Shanghai Institute of Biochemistry and Cell Biology, Center for Excellence in Molecular Cell Science, Chinese Academy of Sciences, Shanghai, China

**Keywords:** anti-aging, canine chronic kidney disease, feline hepatitis, mesenchymal stem cells, Muse cells

## Abstract

**Introduction:**

Multilineage-differentiating stress-enduring (Muse) cells, a subpopulation of mesenchymal stem cells (MSCs) marked by stage-specific embryonic antigen 3 (SSEA3), exhibit superior regenerative capacity compared to conventional MSCs, including enhanced tissue homing, pluripotency, and paracrine effects. However, their natural scarcity (1–5% in MSC populations) limits therapeutic scalability. In this study, we developed a five-compound small molecule method to obtain compound-enriched Muse-like MSCs and assessed their potential use in treating severe veterinary chronic diseases, such as hepatitis and chronic kidney disease (CKD).

**Methods:**

Umbilical cord-derived MSCs from cats and dogs were isolated and cultured, using a combination of five small molecules to obtain enriched Muse-like cells. The cultivation process was verified by immunofluorescence and flow cytometry. Differentiation potential of obtained Muse-like cells was evaluated under lineage-specific conditions. These compound-enriched Muse-like MSCs were administered intravenously (2 × 10^6^ cells/kg) to a 6-year-old cat with severe hepatitis (twice a week for 2 weeks) and a 16-year-old dog with CKD (weekly for 4 weeks). Serum biochemistry and clinical observations were monitored pre- and post-treatment.

**Results:**

Screening of over 100 small-molecule compounds identified optimized five-compound cocktails—valproic acid (0.5 mM), CHIR99021 (3 μM), PD0325901 (0.5 μM), Trolox (10 μM), and nicotinamide (1 mM) for feline MSCs; parnate (10 μM), CHIR99021 (3 μM), PD0325901 (0.5 μM), Trolox (10 μM), and Y27632 (10 μM) for canine MSCs. These small-molecule cocktail effectively boosted SSEA3 positivity, from 0.1–1% to approximately 40%, as confirmed by immunofluorescence staining and flow cytometry. These enriched Muse-like cells demonstrated superior stress tolerance and robust spontaneous differentiation into endodermal (KRT7 + hepatocyte-like), mesodermal (cTnI+ cardiomyocyte-like), and ectodermal (Nestin+ neural progenitor) lineages under targeted induction conditions, which were not observed in untreated MSCs. In therapeutic applications, the enriched Muse-like MSCs normalized feline liver indices by day 21 and improved canine renal markers by day 28, accompanied by notable anti-aging effects.

**Conclusion:**

A small molecule cocktail method was introduced to enhance the Muse population in MSCs, which provide a safe and effective way to harvest Muse cells. These Muse-like MSCs demonstrate high clinical potential for chronic and age-related degenerative diseases, making it a safe and effective therapeutic option.

## 1 Introduction

Multilineage-differentiating stress-enduring (Muse) cells are a distinct subpopulation of mesenchymal stem cells (MSCs). They are mainly characterized by the expression of stage-specific embryonic antigen 3 (SSEA3). Initially identified in human bone marrow and dermal fibroblasts, Muse cells act as endogenous, non-tumorigenic pluripotent stem cells. They can differentiate into cells of all three germ layers without genetic manipulation (1, 2). Muse cells also possess unique biological features, including high stress tolerance, efficient homing to damaged tissues via a number of factors (sphingosine-1-phosphate, SDF-1 and HGF) (3–6), and spontaneous differentiation into tissue-specific cells. Their potent paracrine effects can effectively trigger anti-inflammatory, immunomodulatory, and regenerative responses (7–9). Compared to ordinary MSCs, Muse cells offer superior therapeutic potential. For instance, they achieve enhanced *in vivo* engraftment rates (80–95% differentiation efficiency) and reduce immunogenicity due to low major histocompatibility complex expression (9). They can persist long-term in tissues and avoid teratoma formation. These advantages make them ideal for regenerative applications in chronic and inflammatory conditions (10, 11).

During last few decades, MSCs have been widely applied in human clinical trials for curing various diseases (12, 13). In recent years, MSCs have also been investigated for various applications in veterinary medicine, such as osteoarthritis, chronic kidney disease, spinal cord injuries, chronic gingivostomatitis inflammatory bowel disease and ocular disorders, to name just a few. However, conventional MSC therapies have limitations, including variable efficacy and low engraftment rates. Comparatively, Muse cells’ pluripotent-like properties and superior homing capabilities could address these issues, potentially leading to greater efficacy (14–16). In human therapeutics, Muse cells have shown promising results for conditions such as stroke, myocardial infarction, and liver disease. Their targeted migration and multilineage differentiation support tissue repair with minimal adverse effects (17). Randomized trials reaffirm their benefits such as reduced inflammation, enhanced tissue repair, and improved clinical outcomes (18–22). Based on these inspiring results, we can likewise anticipate the potential advantages of applying Muse cells in veterinary therapeutics.

Given all the merits of Muse cells, a key challenge for their broader therapeutic application is that Muse cells naturally comprise only 0.1–5% of MSC populations. This scarcity limits their functionality and therapeutic scalability (14). To address this challenge, we have performed screening of a variety of small-molecule compounds and identified a five-component combination that, when added to the culture medium, significantly increased the proportion of Muse cells in feline and canine MSCs, from baseline levels of 0.1–1% to around 40%. We then applied these enriched Muse-like MSCs to treat chronic diseases in geriatric animals, such as severe hepatitis and CKD and demonstrated the strong potential of Muse cells in veterinary regenerative medicine.

## 2 Materials and methods

### 2.1 Isolation and culture of feline and canine umbilical cord-derived MSCs

Umbilical cords were collected aseptically from healthy feline and canine neonates following natural delivery. The collected tissue was washed three times with phosphate-buffered saline containing 1% Penicillin–Streptomycin (PS) to remove blood clots. The cleaned umbilical cord was minced into 1–2 mm fragments using sterile scissors and digested with 0.1% collagenase type I (Gibco) in HBSS at 37 °C for 1 h with gentle agitation. The digested content was neutralized using Dulbecco’s modified Eagle’s medium (DMEM; Gibco) containing 10% fetal bovine serum (FBS; Gibco), filtered through a 70-μm cell strainer (JET), and centrifuged at 500 × g for 10 min. The obtained cell pellet was resuspended in DMEM supplemented with 10% FBS and 1% phosphate-buffered saline (PBS) and seeded at a density of 1 × 10^4^ cells/cm^2^ in T-75 flasks (Corning). Cultures were maintained at 37 °C in a humidified atmosphere with 5% CO_2_, with medium changed every 2–3 days. Upon reaching 80% confluency, cells were detached using 0.25% trypsin–EDTA (Gibco) and subcultured at a 1:3 ratio. Finally, cells from passages 3–5 were used for subsequent experiments.

### 2.2 Compound screening and enrichment of muse-like cells

To enrich SSEA3-positive Muse-like cells in isolated feline and canine MSCs, a library of over 100 small-molecule compounds was screened for their ability to upregulate SSEA3 expression (Supplementary data 1). The screening process began by individually adding each compound to compare its effect on increasing SSEA3 positivity rates, followed by combining those compounds that showed improvements in positivity rates, and ultimately selecting the optimal compound combinations. Those compounds, including epigenetic modifiers, signaling pathway inhibitors, and metabolic regulators, were tested both individually and in combinations at varying concentrations in DMEM supplemented with 10% FBS and 1% PS. MSCs at passages 3–5 were treated with these set recipes for 96 h. SSEA3 expression in different sets was quantified by flow cytometry (as described in Section 2.6). After optimization among various combinations, five-compound cocktails were identified: for feline MSCs, 5C: valproic acid 0.5 mM, CHIR99021 3 μM, PD0325901 0.5 μM, Trolox 10 μM, and Nicotinamide 1 mM; for canine MSCs, 5C: parnate 10 μM, CHIR99021 3 μM, PD0325901 0.5 μM, Trolox 10 μM, and Y27632 10 μM. Muse-like MSCs and untreated MSCs were used for differentiation assays for comparison. Enriched Muse-like MSCs were used for veterinary therapeutic applications (Small molecules in these libraries were purchased from Sigma, Tocris Bioscience, Merck, Cayman, Selleckchem, and Stemgent).

### 2.3 Neural progenitor cell differentiation (23) (ectodermal lineage)

For spontaneous differentiation into neural-like cells, 20,000 cells/cm^2^ are plated in poly-HEMA-coated wells and cultured in STEMdiff™ SMADi Neural Induction Kit (STEMCELL Technologies) at 37 °C, 5% CO_2_ for 7–10 days, with medium additions every 3 days. Then transferred to 0.1% Matrigel(corning)-coated wells for 1–2 weeks of adherent culture in the same medium, allowing outgrowth and evaluation via immunocytochemistry for Nestin.

### 2.4 Cytokeratin 7-positive cell differentiation (24, 25) (endodermal lineage)

For spontaneous differentiation into cytokeratin 7 (CK7)-positive hepatocyte-like cells, 20,000 cells/cm^2^ are plated in poly-HEMA-coated wells with low-glucose DMEM containing 10% FBS (supplemented with 1 ng/mL FGF-2) for 7–10 days in suspension at 37 °C, 5% CO_2_ to form clusters. These are adhered on gelatin-coated surfaces for 1–2 weeks in the same medium. Endodermal markers like CK7 were tested to identify successful differentiation.

### 2.5 Cardiomyocyte-like cell differentiation (23, 24) (mesodermal lineage)

For spontaneous differentiation into cardiomyocyte-like cells, 20,000 cells/cm^2^ are treated in suspension with 10 μM 5′-azacytidine in low-glucose DMEM (10% FBS, 2 ng/mL bFGF) for 3 days in poly-HEMA-coated plates at 37 °C, 5% CO_2_. Then transferred to laminin-coated dishes overnight, induced for 7 days with TGF-β1 (2.5 ng/mL), BMP-4 (5 ng/mL each), activin A (10 ng/mL), Wnt-3a (50 ng/mL), and bFGF (10 ng/mL) in 2% FBS medium, followed by 8–21 days with TGF-β1 (2.5 ng/mL), IGF-1 (5 ng/mL), HGF (20 ng/mL), and cardiotrophin-1 (200 ng/mL). Evaluation of markers, such as cTnI, is carried out after 3 weeks.

### 2.6 Flow cytometry analysis

fMSCs and cMSCs were dissociated with TrypLE Express (Gibco) and washed with PBS. Cells were then incubated with 1:200 rat anti-SSEA3 (Invitrogen) at 4 °C overnight. Secondary staining was performed with a secondary antibody conjugated to Alexa Fluor 488 (1:1,000; Invitrogen) for 1 h at room temperature. Samples were analyzed with an ACEA NovoCyte flow cytometer. A minimum of 10,000 events were acquired per sample. Data was processed with ACEA NovoExpress software, and the percentage of SSEA3-positive cells was determined relative to the isotype control.

### 2.7 Immunofluorescence staining

For detection of stage-specific embryonic antigen 3 (SSEA3), cardiac troponin I (cTnI), keratin 7 (KRT7), and Nestin, cells were seeded on glass coverslips in 24-well plates at 5 × 10^3 cells/well and cultured for 24 h. Cells were then fixed with 4% paraformaldehyde (PFA; Betyotime) in PBS for 15 min at room temperature, permeabilized with 0.1% Triton X-100 (Betyotime) for 10 min, and blocked with 5% bovine serum albumin (BSA; Sigma-Aldrich) in PBS for 1 h. Primary antibodies were applied overnight at 4 °C as follows: anti-SSEA3 (rat monoclonal, 1:200; Abcam), anti-cTnI (mouse monoclonal, 1:200; Invitrogen), anti-KRT7 (mouse monoclonal, 1:100; Abcam), and anti-nestin (mouse monoclonal, 1:200; Abcam). After washing three times with PBS, cells were incubated with appropriate Alexa Fluor 488-conjugated secondary antibodies (goat anti-rat IgM or goat anti-mouse IgG, 1:500; Invitrogen) for 1 h at room temperature in the dark. Nuclei were counterstained with 4′,6-diamidino-2-phenylindole (DAPI; 1 μg/mL; Sigma-Aldrich) for 5 min. Coverslips were mounted with ProLong Gold antifade reagent (Invitrogen) and imaged using a confocal laser scanning microscope (Nikon).

### 2.8 Serum biochemical analysis

Vein blood samples (2 mL) were collected from treated cat and dog using separator tubes pre- and post-treatment for biochemical analysis. Samples were first kept at room temperature for 30 min to allow clotting and then centrifuged at 1,500 × g for 10 min to obtain serum. Additional blood samples (1 mL) were collected with EDTA-anticoagulated tubes for hematological analysis. Hematological Analyses were performed using the ProCyte One hematology analyzer (five-part differential) and biochemical parameters were tested using Catalyst One fully automated multifunctional analyzer.

### 2.9 Clinical trial protocol for Muse-like stem cells transplantation

The evaluation of therapeutic efficacy of Muse-like cells primarily relies on self-comparison of selected patients before and after cell therapy. The selected cases had all undergone conventional treatments prior to receiving cell therapy, with no improvement or even deterioration in their conditions. The results of blood biochemical tests after conventional treatments serve as the baseline condition before cell therapy, designated as day 0.

The selected animals, which had shown no significant improvement after observation under conventional drug treatments, were administered Muse-like cells intravenously at a uniform dose of 2 × 10^6^ cells/kg body weight for both cats and dogs. After receiving cell therapy, blood samples are collected sequentially at different intervals depending on the disease being treated: for feline liver disease, samples are collected every 7 days (day 7, day 14, day 21, etc.); for canine kidney disease, samples are collected every 14 days (day 14, day 28, etc.). A comparison of these indicators before and after cell therapy is used as an effective self-control and efficacy assessment.

### 2.10 Statistical analysis

Data are presented as mean ± SD. Statistical analyses were performed using GraphPad Prism software (version 10.0). For multiple group comparisons with one independent variable, one-way ANOVA followed by Tukey’s *post hoc* test was used. For datasets involving two independent variables, two-way ANOVA followed by Tukey’s or Sidak’s post hoc test was applied as indicated in the figure legends. Unpaired two-tailed Student’s t-test was used for direct comparisons between two groups where appropriate. Exact *p* values and specific tests applied are indicated in the respective figure legends.

## 3 Results

### 3.1 Enrichment of SSEA3 expression in feline umbilical cord-derived MSCs

**Figure 1:**
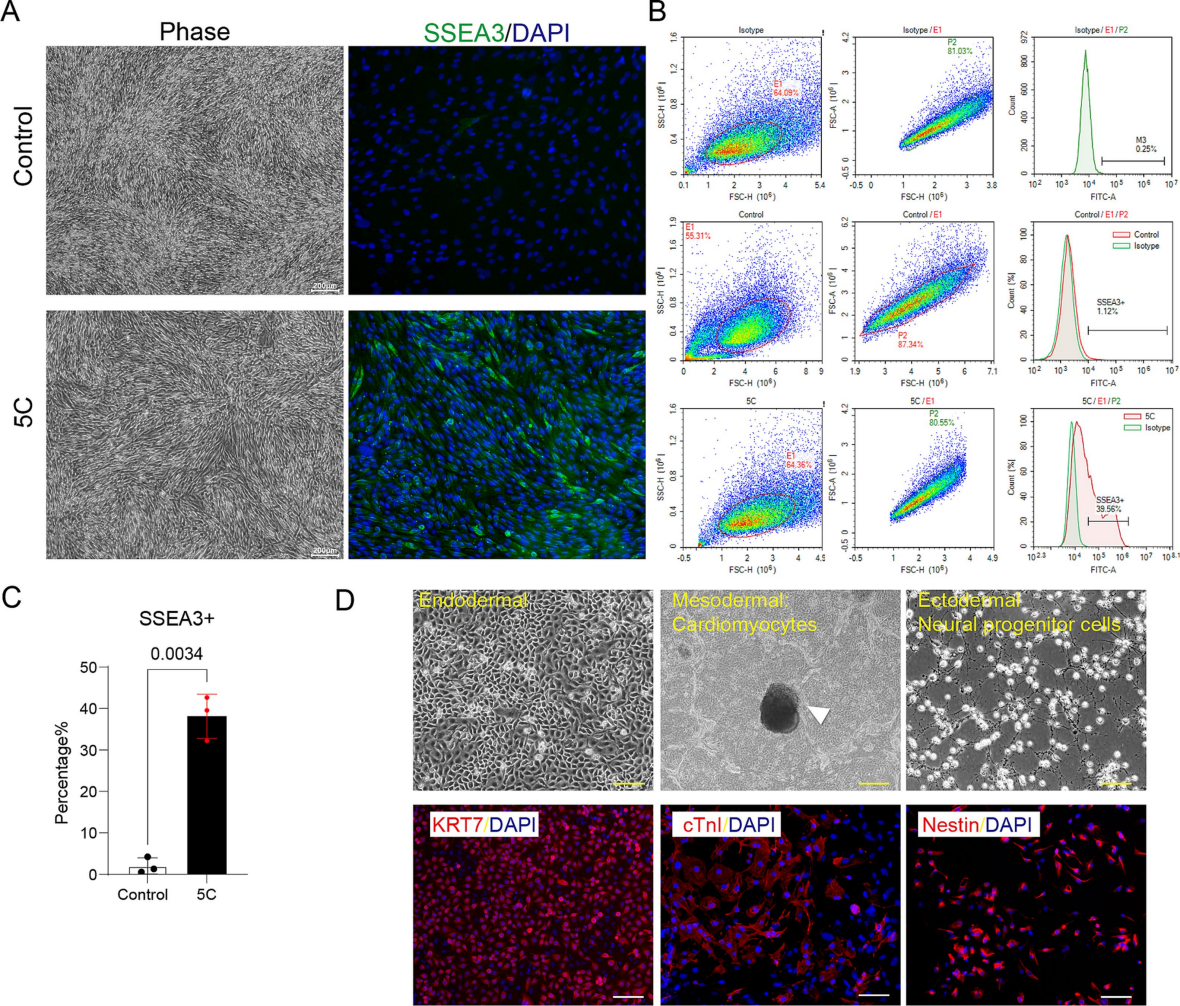
Enrichment and characterization of Muse-like cells in feline umbilical cord-derived MSCs. **(A)** Phase-contrast images (left) showing spindle-shaped morphology in control and 5C-treated groups, alongside immunofluorescence staining (right) for SSEA3 (green) with DAPI nuclear counterstain (blue), revealing minimal SSEA3 positivity in controls versus intense, widespread expression post-5C enrichment. Scale bar: 200 μm. **(B)** Flow cytometry histograms quantifying SSEA3-positive rates. **(C)** Bar graph summarizing the statistical analysis of SSEA3-positive cell percentages from flow cytometry data, presented as mean ± SD (*N* = 3). **(D)** Differentiation potential under lineage-specific conditions of Muse-like cells: phase-contrast images (top) of endodermal clusters and mesodermal cardiomyocyte aggregates (arrowhead), with immunofluorescence (bottom) confirming KRT7 (keratin 7, red, endodermal), cTnI (cardiac troponin I, red, cardiomyocyte), and Nestin (red, neural progenitor cells) positivity co-localized with DAPI (blue). Scale bar: 100 μm.

To identify an optimal strategy for enriching Muse cells in feline umbilical cord-derived MSCs, we conducted a systematic screening of a library comprising over 100 small-molecule compounds known for their roles in stem cell modulation, including inhibitors of epigenetic modifiers, signaling pathway regulators, and metabolic activators (Supplementary data 1). Through iterative dose–response assays and viability assessments, a combination of five compounds (5C: valproic acid 0.5 mM, CHIR99021 3 μM, PD0325901 0.5 μM, Trolox 10 μM, and Nicotinamide 1 mM)—selected for their synergistic effects on pluripotency markers without inducing cytotoxicity—was identified as the most effective in upregulating SSEA3 expression, a hallmark of Muse cells. Phase-contrast imaging confirmed that both control and 5C-treated feline MSCs maintained a characteristic spindle-shaped, fibroblast-like morphology, indicating healthy mesenchymal phenotype (Figure 1A). However, immunofluorescence staining result indicated a stark difference: control MSCs showed only sparse green fluorescent signals for SSEA3 amidst DAPI-stained nuclei, reflecting a low baseline Muse cell content, whereas 5C-treated cells exhibited intense and widespread green fluorescence, demonstrating substantial enrichment of SSEA3-positive Muse-like cells (Figure 1A). Quantitative validation via flow cytometry further corroborated this, with control groups displaying minimal SSEA3 positivity (1.12%), this value escalated significantly to 39.56% following 5C treatment (*p* = 0.0034,as quantified in the bar graph; Figures 1B,C). Furthermore, the enriched Muse-like cells also expressed the mesenchymal stem cell marker CD105 but not the hematopoietic marker CD117, consistent with the characteristics of standard Muse cells (Supplementary Figure S1). Moreover, to evaluate their stress-enduring capacity, a defining trait of Muse cells, the enriched population and untreated MSCs were both subjected to 12-h enzymatic digestion followed by AOPI viability assessment. Muse-like cells demonstrated significantly higher survival rates than untreated MSCs (*p* = 0.0024), underscoring their enhanced tolerance to proteolytic stress (Supplementary Figures S2A,C). Under lineage-specific differentiation protocols, the 5C-enriched Muse-like MSCs readily differentiated into endodermal lineages, as evidenced by robust red fluorescence for KRT7 (a marker of epithelial/endodermal commitment),mesodermal lineages, forming cardiomyocyte-like aggregates (arrowhead-indicated) with positive red staining for cTnI (cardiac troponin I; Figure 1D and Supplementary Video S1), and ectodermal lineages, differentiating into Nestin-positive neural progenitor cells (NPCs). In contrast, untreated control MSCs exhibited no morphological adaptations or marker expression (data not show), showing a complete absence of differentiation trends even under identical induction conditions, further confirming the pluripotent-like superiority of Muse-enriched populations in enabling multilineage potential without genetic manipulation.

### 3.2 Enrichment of SSEA3 expression in canine umbilical cord-derived MSCs

**Figure 2:**
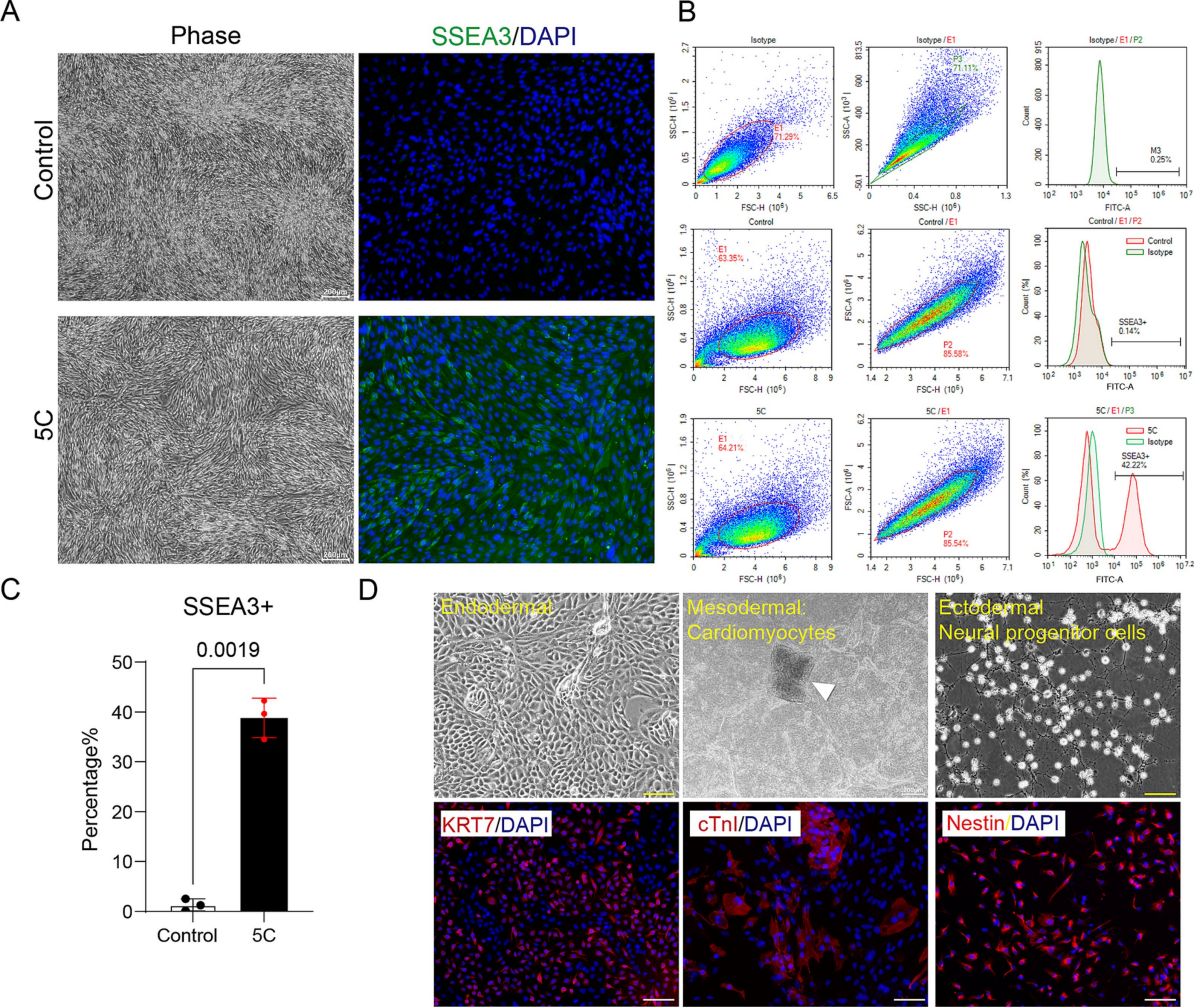
Enrichment and characterization of Muse-like cells in canine umbilical cord-derived MSCs. **(A)** Phase-contrast images (left) showing spindle-shaped morphology in control and 5C-treated groups, alongside immunofluorescence staining (right) for SSEA3 (green) with DAPI nuclear counterstain (blue), revealing minimal SSEA3 positivity in controls versus intense, widespread expression post-5C enrichment. Scale bar: 200 μm. **(B)** Flow cytometry histograms quantifying SSEA3-positive rates. **(C)** Bar graph summarizing the statistical analysis of SSEA3-positive cell percentages from flow cytometry data, presented as mean ± SD (*N* = 3). **(D)** Differentiation potential under lineage-specific conditions of Muse-like cells: Phase-contrast images (top) of endodermal clusters and mesodermal cardiomyocyte aggregates (arrowhead), with immunofluorescence (bottom) confirming KRT7 (keratin 7, red, endodermal), cTnI (cardiac troponin I, red, cardiomyocyte), and Nestin (red, neural progenitor cells) positivity co-localized with DAPI (blue). Scale bar: 100 μm.

Based on the same screening paradigm applied to feline MSCs, we focused on exploring compounds that could selectively amplify Muse cell fractions in canine umbilical cord-derived MSCs while preserving cell integrity. After multiple rounds of combinatorial testing, including high-throughput viability screening and marker expression analyses, the five-compound cocktail (5C: parnate 10 μM, CHIR99021 3 μM, PD0325901 0.5 μM, Trolox 10 μM, and Y27632 10 μM) standed out, exhibiting cross-species efficacy in boosting SSEA3 levels through targeted modulation of stemness pathways. Phase-contrast microscopy revealed consistent fibroblast-like morphology in both control and 5C-treated canine MSCs, with no overt signs of stress or alteration (Figure 2A). Immunofluorescence staining, however, delineated clear enrichment: control groups displayed faint and infrequent green SSEA3 signals, while 5C-treated cells showed robust, abundant green fluorescence, signifying a substantial rise in Muse-like cell content (Figure 2A). Flow cytometry provided precise quantification, confirming low baseline SSEA3 positivity in controls (0.14%), and much higher positivity (42.22%) post-5C treatment (*p* = 0.0019,as quantified in the bar graph; Figures 2B,C). Additionally, these enriched Muse-like cells showed positive staining for the MSC marker CD105 and negative staining for CD117, reinforcing their Muse cell identity (Supplementary Figure S1). Further assessment of stress tolerance revealed that canine Muse-like cells maintained superior viability after 12-h enzymatic digestion compared to untreated MSCs (*p* = 0.0020), as quantified by AOPI staining (Supplementary Figures S2B,C). In differentiation assays, 5C-enriched Muse-like MSCs demonstrated versatile potential under tailored conditions, successfully forming endodermal structures with strong red KRT7 positivity, mesodermal cardiomyocytes exhibiting cTnI fluorescence in distinct aggregates (Figure 2D and Supplementary Video S2), and ectodermal lineages, differentiating into Nestin-positive neural progenitor cells (NPCs). Untreated canine MSCs, conversely, lacked any detectable marker expression or structural changes (data not shown), indicating negligible differentiation capacity and reinforcing the critical role of Muse enrichment in unlocking advanced regenerative capabilities.

### 3.3 Case study 1: Muse-like MSCs treating severe feline hepatitis

The patient was a 6-year-old spayed female Chinese domestic short-haired cat diagnosed with severe hepatic lipidosis. The clinical symptoms include persistent anorexia, vomiting, salivation, marked jaundice, and lethargy. Initial clinical findings included elevated liver enzymes [ALT at 285 U/L (28–109 U/L), ALP at 580 U/L (11–49 U/L), TBIL at 7.3 mg/dL (0–0.1 mg/dL)]; AST not initially reported (17–48 U/L), hypokalemia(3.8–5.5 mEq/L) and anemia [HGB at 10 g/dL (10.9–15.7 g/dL)]. Abdominal ultrasound confirmed enhanced hepatic echogenicity.

**Figure 3:**
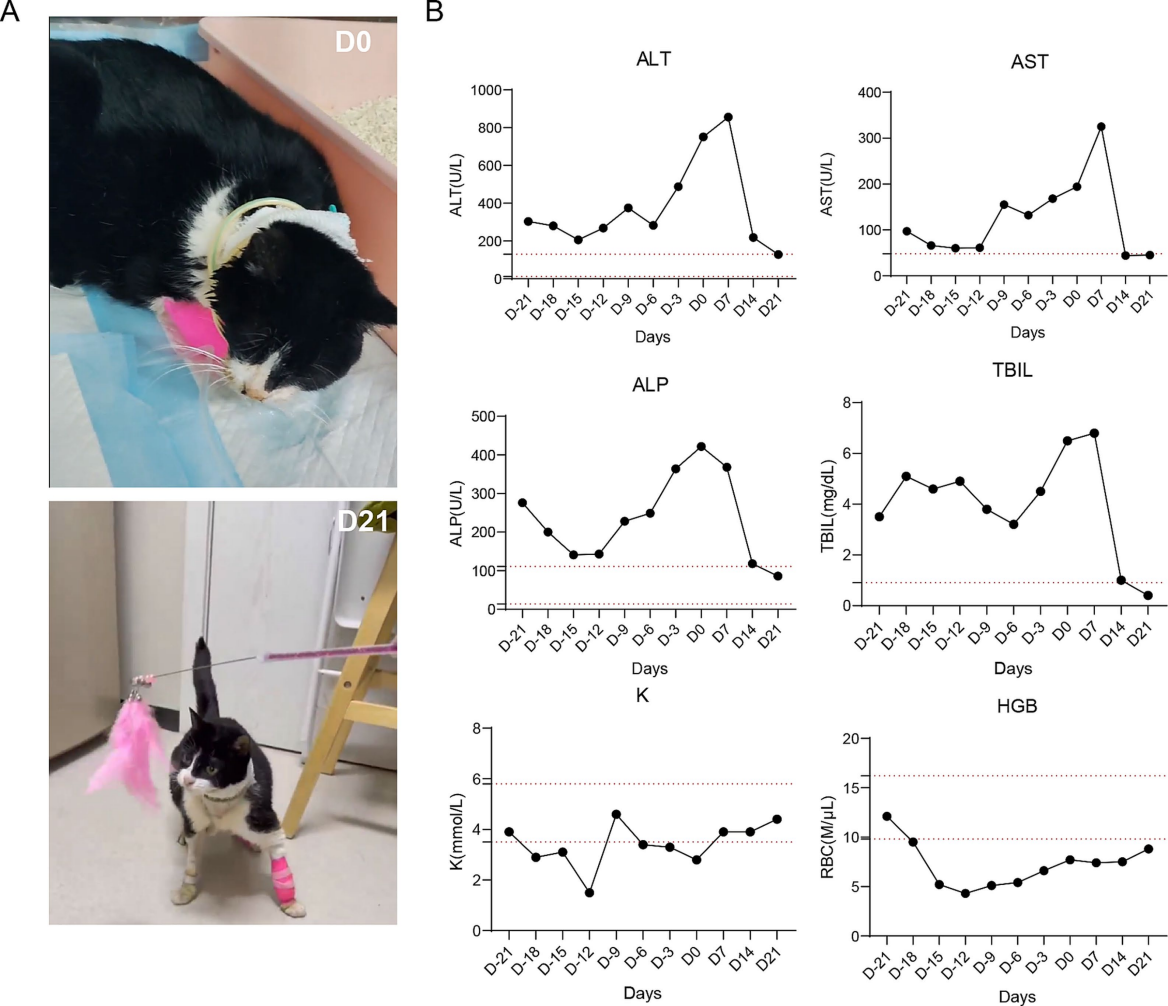
Clinical and biochemical outcomes of Muse-like MSC therapy in a cat with severe hepatitis. **(A)** Photographs depicting the cat’s condition at day 0 (D0: lethargic, jaundiced, requiring supportive care) and day 7 (D7: active, alert, with restored vitality and independent behavior). **(B)** Line graphs tracking serum biomarkers over 21 days: ALT (U/L) peaking then declining to near-normal; AST (U/L) showing similar resolution; ALP (U/L) reducing progressively; TBIL (mg/dL) normalizing from elevated levels; K (mmol/L) stabilizing with minor fluctuations; HGB (g/dL) indicating gradual anemia alleviation. Dashed red lines represent reference ranges.

Before administration of Muse-like cell therapy, this patient received regular treatment starting 2 months prior, including intravenous fluids, esophagostomy tube feeding, antiemetics (maropitant), antibiotics (metronidazole), hepatoprotectants (Livertech, ursodeoxycholic acid), and vitamin B12. However, the biochemical parameters deteriorated (ALT peaking at 751 U/L) and clinical symptoms worsened, including ongoing lethargy, anorexia, salivation, and jaundice (as shown in Figure 3A, D0).

Following the ineffectiveness of standard treatments, the cat received intravenous administration of 5C-enriched Muse-like mesenchymal stem cells (MSCs) at a dose of 2 × 10^6^ cells/kg body weight, given twice weekly for 2 weeks. Recovery was rapid and comprehensive, with initial fluctuations in some biochemical parameters followed by steady improvements, leading to complete resolution of hepatic lipidosis by day 21. Both clinical indices and behavioral changes progressed positively over the timeline. 7 days after cell therapy, anemia began to recover, with HGB rising to ~11 g/dL, and salivation ceased. By day 14, marked declines in biochemical markers were observed: ALT dropped to 219 U/L, AST to 44 U/L, ALP to 118 U/L, and TBIL to 1.0 mg/dL, approaching reference ranges. HGB improved to ~12 g/dL, correlating with reduced fatigue and better mucous membrane color. Clinically, the cat also showed improved vitality, decreased lethargy and jaundice. By day 21, full normalization occurred: ALT reached 129 U/L, AST 45 U/L, ALP 86 U/L, and TBIL 0.4 mg/dL, all within reference ranges. HGB stabilized at ~13 g/dL, indicating alleviation of anemia. Behaviorally, the cat exhibited complete recovery of mobility and vitality, transitioning from a lethargic state (Figure 3B, D0) to active, independent behavior with normal grooming and exploration (Figure 3B, D21). Appetite returned spontaneously, allowing removal of the esophagostomy tube, and autonomous feeding, drinking, without other complications (as documented in Supplementary Video S3).

### 3.4 Case study 2: Muse-like MSCs treating canine chronic kidney disease

**Figure 4:**
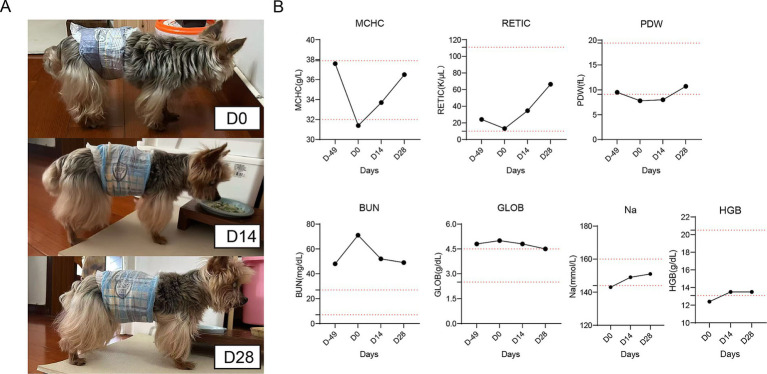
Therapeutic response to Muse-like MSCs in an elderly dog with chronic nephritis. **(A)** Photographs illustrating progression at D-49, D0 (hunched, incontinent, poor coat), D14 (improved posture, increased activity), and D28 (upright stance, glossy coat, no diapers needed). **(B)** Line graphs of hematological and renal parameters over 28 days: MCHC (g/dL), RETIC (K/μL), PDW (fL), Na (mmol/L), SDMA (μg/dL), BUN (mg/dL), GLOB (g/dL), and HGB (g/dL), demonstrating steady improvements toward normalization. Dashed red lines denote reference ranges.

The patient was a 16-year-old neutered male Yorkshire Terrier diagnosed with advanced chronic kidney disease (CKD), characterized by long-term abnormal renal indicators. The dog also exhibited a range of debilitating symptoms, including poor mobility, a hunched back, severe hair loss, progressive weight loss, lethargy, and reduced responsiveness (see Figure 4A).

Initial blood analysis on day 0 confirmed marked azotemia and associated abnormalities: blood urea nitrogen (BUN) at 71 mg/dL (reference range: 7.0–27.0 mg/dL), symmetric dimethylarginine (SDMA) at 21 μg/dL (0–14 μg/dL), Globulin (GLOB) at 5 g/dL (2.5–4.5 g/dL), hemoglobin (HGB) at 12.4 g/dL (13.1–20.5 g/dL), and mean corpuscular hemoglobin concentration (MCHC) at 31.4 g/dL (32–37.9 g/dL). Additional anomalies included a low reticulocyte count (RETIC) at 13.1 K/μL (10–111 K/μL), platelet distribution width (PDW) at 7.8 fL (9.1–19.4 fL), and slightly low sodium (Na) at 143 mmol/L (144–160 mmol/L) (Figure 4B).

The dog then received Muse-like mesenchymal stem cell therapy through intravenous administration (2 × 10^6^ cells/kg body weight) weekly for 4 consecutive weeks. Close monitoring was conducted for potential adverse events, such as infusion reactions or immunological responses, and no significant complications occurred during the therapy period.

Recovery was progressive, with notable improvements in both clinical indices and behavioral aspects observed over the timeline. By day 7, early signs of recovery emerged, and the dog displayed increased activity, improved appetite, and better posture while eating. By day 14, renal markers continued to decline: SDMA dropped to 16 μg/dL and BUN decreased from 71 mg/dL to 52 mg/dL. GLOB reduced to 4.8 g/dL. Anemia-related indices showed positive shifts, including MCHC rising to 33.7 g/dL, RETIC increasing to 34.6 K/μL (suggesting improved erythropoiesis), PDW normalizing to 8 fL, Na stabilizing at 149 mmol/L, and HGB improving slightly to 13.5 g/dL. Behaviorally, aging symptoms lessened, with the hunched back started straightening, improved urinary control, less hair loss, and heightened responsivity to stimuli (as documented in Supplementary Video S4). By day 28, substantial recovery in CKD markers and partial reversal of aging features were observed. SDMA reached 15 μg/dL, within the normal range, while BUN lowered further to 49 mg/dL and GLOB normalized at 4.5 g/dL. Hematological improvements included MCHC climbing to 36.5 g/dL, RETIC surging to 66.4 K/μL (reflecting boosted red blood cell production), PDW at 10.7 fL, Na at 151 mmol/L. Behaviorally, severe hair loss minimized to normal levels, more agile movements were observed, and a rejuvenated appearance with enhanced vitality was achieved.

## 4 Discussion

The use of small molecules to enrich Muse-like stem cells from mesenchymal stem cells (MSCs) derived from feline and canine umbilical cord tissue offers several key advantages, primarily centered on safety, scalability, and avoidance of complex manipulations (26–28). This protocol avoids genetic engineering, which reduces potential risks associated with viral vectors or other invasive techniques that could introduce oncogenic mutations or immunogenicity issues (6, 29). Instead, it relies on non-genetic, chemical-based enrichment, making it a safer and more straightforward approach for clinical application in veterinary medicine.

The efficiency of this small-molecule enrichment protocol, referred to as the 5C method in our study, is particularly noteworthy, as it significantly boosts the proportion of SSEA3-positive Muse-like cells to 40%. This represents a substantial improvement over baseline levels (0.1–1%), enabling higher yields that address scalability challenges in regenerative therapies. Such enrichment facilitates the production of sufficient cell quantities for therapeutic dosing in a single preparation, which is crucial for treating larger animals or multiple cases. Preclinical data from models of hepatic injury and renal ischemia further validate this efficiency, showing sustained efficacy without long-term complications, and aligning with evidence from large animal studies where Muse cells achieved engraftment rates of 80–95%, far surpassing standard MSCs (15, 30, 31).

In veterinary clinical trials, Muse cells demonstrate promising therapeutic potential, particularly for chronic conditions like severe hepatitis and chronic kidney disease (CKD), where traditional treatments often fall short (30, 32, 33). In the feline hepatitis case, intravenous administration of enriched Muse-like cells led to complete resolution of hepatic dysfunction within 4 weeks, with liver enzymes normalizing dramatically (e.g., ALT from 856 U/L to 129 U/L; AST from 325 U/L to 44 U/L), jaundice resolving, and full recovery of appetite and vitality. This outperforms standard therapies such as antibiotics, anti-inflammatories, and fluid support, which frequently result in recurrence rates over 50% and limited long-term survival. In the canine CKD case, Muse cell infusion over 4 weeks reduced key renal markers (e.g., SDMA from 21 μg/dL to 15 μg/dL; BUN from 71 mg/dL to 49 mg/dL), while also addressing comorbidities such as anemia (RETIC increased from 13.1 to 66.4%) and muscle wasting. This goes beyond the modest improvements typically seen with dialysis or symptomatic management.

Muse cells achieve this through homing to damaged tissues via sphingosine-1-phosphate signaling, spontaneous differentiation, and secretion of anti-inflammatory factors like TGF-β1, leading to rapid antifibrotic effects, reduced collagen deposition, and improved tissue architecture (3, 22). Their stress tolerance and low immunogenicity enable allogeneic use without immunosuppression, providing a prospective therapy to thoroughly cure severe chronic diseases.

Beyond disease-specific repair, Muse cells offer broader rejuvenating and anti-aging effects, especially in geriatric conditions, which comprise over 40% of veterinary cases and often experience multifactorial decline (34, 35). In our case studies, treatment on the old dog not only alleviated CKD, but also revitalized activity, normalized hair growth, and modulated senescence-associated secretory phenotypes. Furthermore, previous researches on animal models confirmed that Muse cells may delay aging-related pathologies, such as degeneration in conditions like osteoarthritis or cognitive dysfunction. Overall, Muse cells’ pluripotent-like properties, high engraftment, and multifaceted benefits—including non-tumorigenic pluripotency, and absence of adverse effects like infusion reactions or ectopic tissue formation—position them as a transformative tool over conventional MSC therapies, which exhibit limited engraftment and variable efficacy in chronic inflammatory conditions. While these case reports provide strong proof-of-concept evidence of the efficacy of Muse cell therapy, more randomized trials to optimize dosing and confirm long-term applications in diverse veterinary scenarios are still needed.

## 5 Conclusion

In conclusion, we developed an optimized five-compound small-molecule method—employing species-specific cocktails (valproic acid 0.5 mM, CHIR99021 3 μM, PD0325901 0.5 μM, Trolox 10 μM, and nicotinamide 1 mM for feline MSCs; parnate 10 μM, CHIR99021 3 μM, PD0325901 0.5 μM, Trolox 10 μM, and Y27632 10 μM for canine MSCs)—to enrich Muse-like MSCs from umbilical cord-derived populations. This protocol substantially elevated SSEA3 positivity from 0.1–1% to approximately 40%, as confirmed by immunofluorescence and flow cytometry, while improving stress tolerance and facilitating robust spontaneous differentiation into endodermal (KRT7 + hepatocyte-like), mesodermal (cTnI+ cardiomyocyte-like), and ectodermal (Nestin+ neural progenitor) lineages—capabilities lacking in untreated MSCs. Therapeutic evaluations in refractory feline hepatitis and canine chronic kidney disease (CKD) validated the enriched cells’ superior tissue repair, immunomodulation, and systemic rejuvenation, with no adverse effects observed. These findings emphasize the safety, efficacy, and clinical potential of Muse-like cells for regenerative applications, advocating for further randomized trials to expand their utility.

## Data Availability

The original contributions presented in the study are included in the article/Supplementary material, further inquiries can be directed to the corresponding author.
