# Supplementary material for: Muse-like stem cell therapy for curing chronic diseases in geriatric feline and canine
Source: Front Vet Sci. 2026 Mar 18;13:1708295. doi: 10.3389/fvets.2026.1708295 (PMC13038508; doi:10.3389/fvets.2026.1708295)
Supplement: Supplementary file 1 [file Data_Sheet_1.PDF]

INVENTORY OF ALL SUPPLEMENTAL ITEMS

- **Supplementary Video S1: Time-lapse footage of 5C-enriched feline Muse-like MSCs undergoing mesodermal differentiation into cardiomyocyte-like aggregates. The video highlights spontaneous formation of beating clusters with positive red fluorescence for cardiac troponin I (cTnI), as observed under lineage-specific induction conditions (related to Figure 1C).**
- **Supplementary Video S2: Time-lapse recording of 5C-enriched canine Muse-like MSCs differentiating into mesodermal cardiomyocyte-like structures. It shows the development of fluorescent aggregates exhibiting cardiac troponin I (cTnI) positivity, confirming multilineage potential under tailored differentiation protocols (related to Figure 2C).**
- **Supplementary Video S3: Clinical observation video of the 6-year-old feline patient with severe hepatitis post-treatment with enriched Muse-like MSCs. The footage documents full recovery by day 21, including normalized eating via esophagostomy tube, autonomous feeding and drinking, resolution of jaundice and salivation, and absence of other complications.**
- **Supplementary Video S4: Post-treatment video of the 16-year-old canine patient with chronic kidney disease (CKD) following administration of enriched Muse-like MSCs. It captures improvements by day 14, such as enhanced urinary control, reduced hair loss, increased mobility and responsivity to stimuli, and overall anti-aging effects, with continued progress noted by day 28.**
- **Supplementary Figure S1. Expression of CD105 and CD117 in enriched Muse-like cells from feline and canine MSCs.**
- **Supplementary Figure S2. Flowchart for small-molecule screening to induce high $SSEA3^+$ Muse-like cells.**
- **Supplementary Figure S3. Enhanced enzymatic stress tolerance of Muse-like cells compared to MSCs.**
- **Supplementary Figure S4. Ultrasonographic comparison of feline liver and gallbladder and canine kidneys pre- and post-cell therapy.**

- **Supplementary Data 1 Small-molecule compounds used in primary screening.**

Supplementary Figure S1. Expression of CD105 and CD117 in enriched Muse-like cells from feline and canine MSCs.

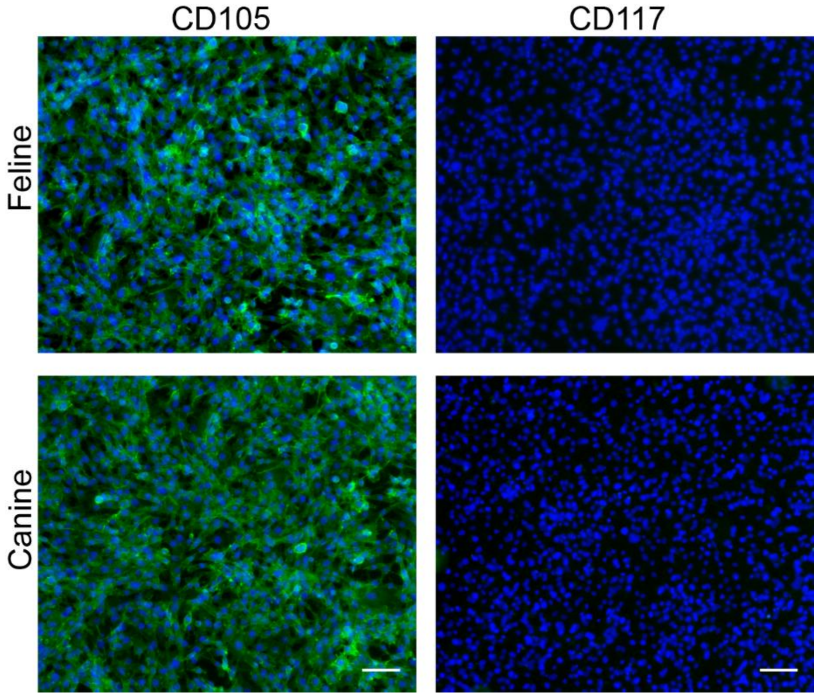

Immunofluorescence staining for CD105 (green, left) and CD117 (green, right) with DAPI nuclear counterstain (blue) in 5C-treated feline MSCs, demonstrating strong CD105 positivity and negligible CD117 expression. Scale bar: 200μm.

Supplementary Figure S2. Flowchart for small-molecule screening to induce high SSEA3^{+} Muse-like cells.

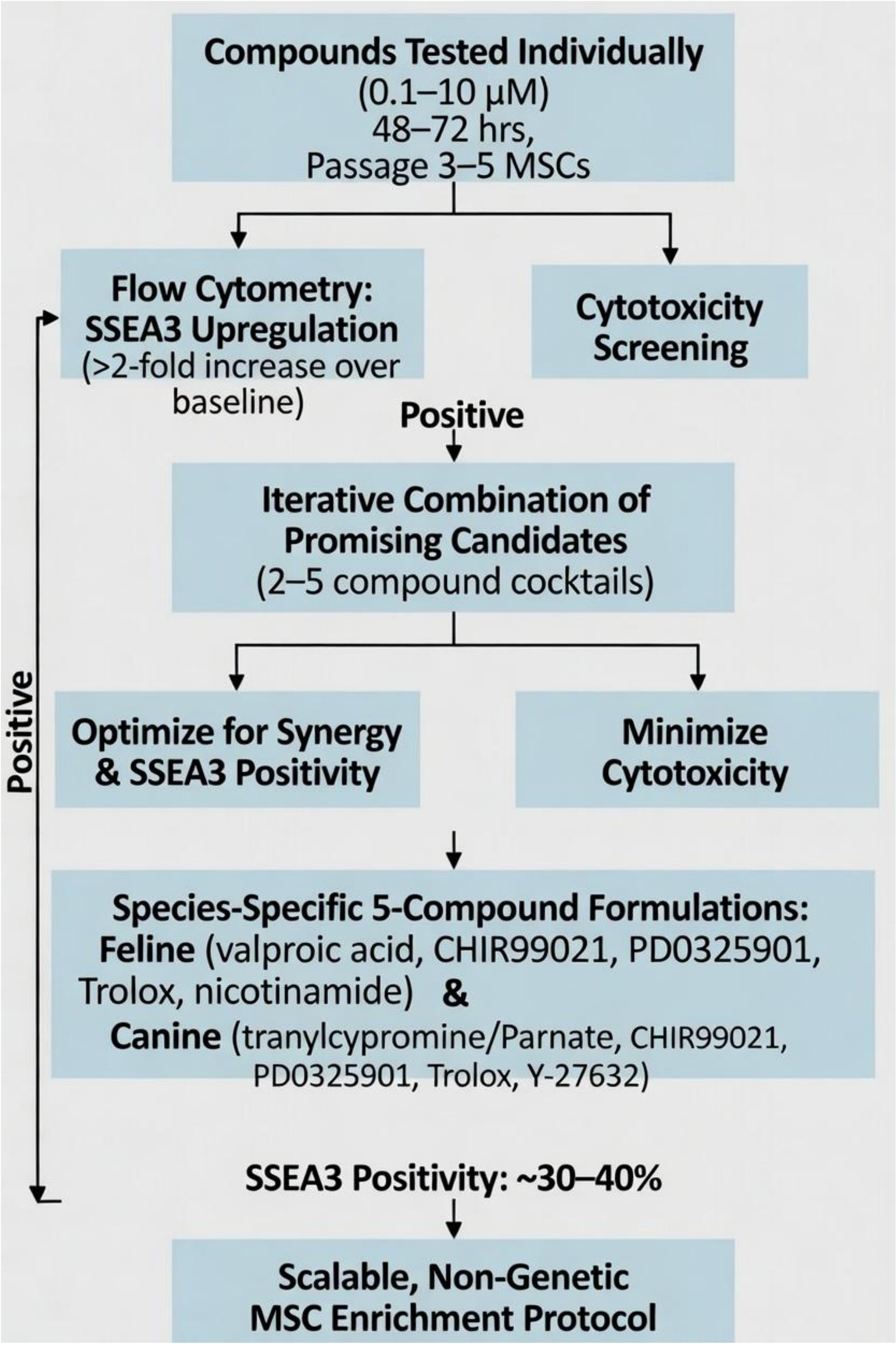

Supplementary Figure S3. Enhanced enzymatic stress tolerance of Muse-like cells compared to MSCs.

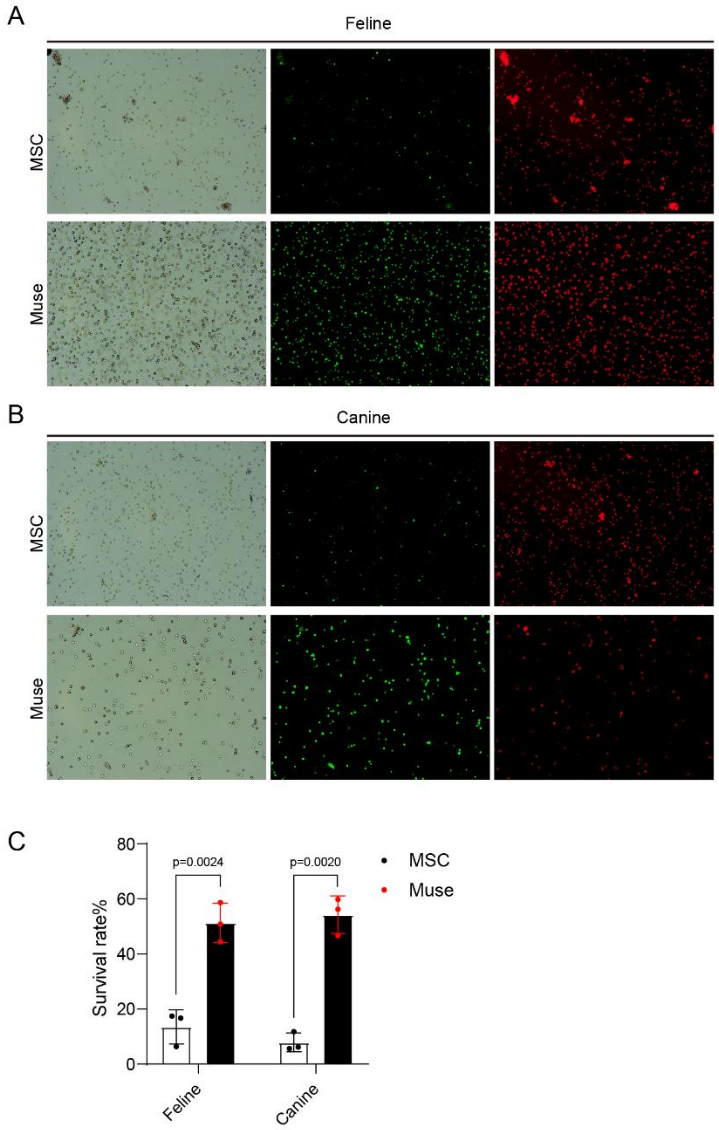

(A) Representative phase-contrast images (left) and AOPI fluorescence staining (right; green: live cells, red: dead cells) of enriched Muse-like cells and control MSCs from feline sources after 12-hour enzymatic digestion, illustrating greater viability in Muse-like cells.

(B) Corresponding phase-contrast and AOPI staining images for canine Muse-like cells and control MSCs post-digestion, showing similar improved survival in the enriched population.

(C) Bar graph summarizing survival rates (mean ± SD, N=3) for feline and canine MSCs versus Muse-like cells, with statistical significance indicated by unpaired t-tests ($p=0.0024$ for feline, $p=0.0020$ for canine).

Supplementary Figure S4. Ultrasonographic comparison of feline liver and gallbladder and canine kidneys pre- and post-cell therapy.

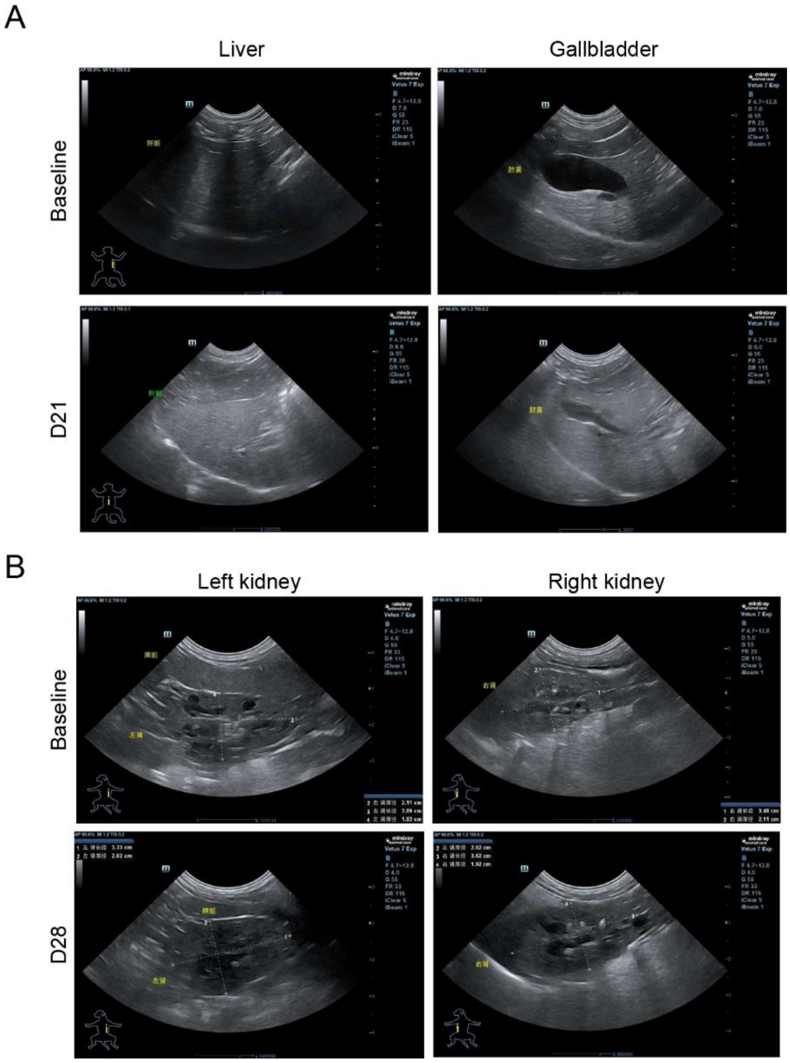

(A) Ultrasonographic images of the liver (left column) and gallbladder (right column) in a cat at baseline (top row) and on day 21 post cell treatment (bottom row).

(B) Ultrasonographic images of the left kidney (left column) and right kidney (right column) in a dog at baseline (top row) and on day 28 post cell treatment (bottom row).
